# Excessive Sodium Intake in Commercial Diet Catering Meal Plans in Poland: Implications for Diet Quality and Chronic Disease Risk

**DOI:** 10.3390/nu18081202

**Published:** 2026-04-10

**Authors:** Dominika Patrycja Dobiecka, Karolina Korzonek, Martyna Falkowska, Kinga Wityńska, Justyna Moskwa, Katarzyna Socha, Sylwia Katarzyna Naliwajko

**Affiliations:** Department of Bromatology, Faculty of Pharmacy with the Division of Laboratory Medicine, Medical University of Białystok, 15-222 Białystok, Poland

**Keywords:** sodium intake, daily food rations, dietary patterns, cardiovascular risk, public health nutrition

## Abstract

**Background/Objectives:** Excessive sodium (primarily from sodium chloride, NaCl) intake remains one of the leading dietary risk factors associated with hypertension, cardiovascular disease, and other chronic health conditions worldwide. Commercial diet catering services providing ready-to-eat daily meal plans have become increasingly popular and are often perceived as nutritionally balanced; however, analytical evidence regarding their actual salt content remains limited. The aim of this study was to evaluate the NaCl content of daily food rations (DFRs) offered by commercial diet catering services in Poland. **Methods:** A total of 120 DFRs representing three dietary patterns (Hashimoto diet, DASH diet, and low-carbohydrate diet) were collected from 40 catering providers. Sodium chloride content was determined using the Mohr titration method. Sodium intake values were estimated by conversion from NaCl equivalents to allow comparison with dietary recommendations. **Results:** The median NaCl content across all analyzed diets was 14.19 g/day (Q1: 10.62 g; Q3: 17.49 g), corresponding to approximately 284% of the World Health Organization recommended maximum intake of 5 g/day of salt. Nearly half of the analyzed DFRs (45.83%) exceeded the recommended intake by more than threefold. Overall, 99.2% of the analyzed DFRs exceeded recommended NaCl intake levels, while 91.9% did not comply with the values declared by manufacturers. DFRs consisting of five meals contained higher NaCl levels than three-meal plans (*p* < 0.0196); however, this difference may be related to variation in total food mass rather than meal frequency, as the number of meals was confounded with diet type. **Conclusions:** These findings suggest that commercially prepared diet catering meals may represent a substantial source of dietary NaCl when used as a primary daily food source. Improved nutritional monitoring, clearer nutrient reporting, and quality control of commercially prepared dietary plans may support public health strategies aimed at reducing NaCl intake.

## 1. Introduction

Increased sodium intake is recognized as a major global health risk and an important contributor to the burden of non-communicable diseases. According to WHO data, the average sodium intake in adults is approximately 4310 mg/day (≈10.8 g/day of salt), which exceeds the recommended level of 2000 mg/day (5 g/day of salt) by more than twofold [1,2]. A similar situation is observed in Poland, where average daily salt intake is estimated at 10–15 g/day [3].

Excessive sodium consumption is associated with increased risk of hypertension, cardiovascular disease, kidney disorders, osteoporosis, gastric cancer, and overall mortality [4,5,6,7,8,9]. Meta-analyses and umbrella reviews confirm that sodium reduction lowers blood pressure and reduces cardiovascular risk, highlighting the importance of limiting sodium intake at the population level [10,11].

Because a large proportion of dietary sodium comes from processed and ready-to-eat foods, public health strategies increasingly focus on monitoring the nutritional quality of commercially prepared meals. The WHO “SHAKE the Salt Habit” initiative and European reformulation programs emphasize the need to reduce sodium content in processed foods and improve consumer awareness [12,13]. However, consumers often underestimate the sodium content of foods perceived as healthy, which makes independent assessment of commercially prepared meals particularly important [14,15].

In recent years, commercial diet catering services (so-called boxed diets) have become increasingly popular in Poland and other European countries. These services are marketed as nutritionally balanced and tailored to specific dietary needs, including weight reduction, medical conditions, or dietary patterns recommended for cardiovascular prevention [16]. One of the most commonly promoted dietary models is the DASH diet, which recommends reduced sodium intake (1500–2300 mg/day) and is considered an effective dietary approach for lowering blood pressure and improving metabolic health [17,18,19,20,21,22,23].

Despite the growing popularity of diet catering services, there is a lack of studies based on direct chemical analysis of sodium chloride content in commercially available DFRs, representing an important gap in current knowledge. Unlike pre-packed foods, catering meals are not fully covered by mandatory nutrition labelling regulations under EU Regulation No 1169/2011, and nutrient declarations provided by manufacturers are not always verified analytically [24,25,26,27]. As a result, consumers who rely on catering diets as their main source of daily nutrition may unknowingly consume excessive amounts of sodium.

Although several studies have evaluated sodium intake at the population level, little is known about the actual sodium chloride content of commercially prepared diet catering meals, particularly in Poland, and whether these meals comply with recommended intake levels and with the values declared by manufacturers. At the European level, ongoing food reformulation strategies aim to reduce NaCl content in processed foods, highlighting the importance of monitoring emerging food service sectors such as diet catering.

The aim of this study was to evaluate the sodium chloride content in DFRs provided by diet catering services in Poland, including three dietary models: Hashimoto, DASH, and low-carbohydrate diets. These diet categories were selected due to their popularity among health-conscious consumers and their differing approaches to sodium intake. The DASH diet emphasizes sodium reduction, the low-carbohydrate diet often includes processed, high-sodium animal products, and the Hashimoto diet is increasingly used by individuals with autoimmune thyroid disorders—a group potentially sensitive to dietary imbalances. Additionally, the study aimed to determine whether these meals comply with recommended sodium chloride intake levels and with the values declared by the manufacturers.

## 2. Materials and Methods

### 2.1. Materials

The study material consisted of 120 whole-day meal plans, referred to as DFRs, offered and delivered by diet catering companies in Poland.

Sampling was conducted starting in October 2023 over a continuous data collection period. Catering services (*n* = 40) operating in Białystok were selected based on service availability and menu transparency. Providers were identified using publicly available rankings, online comparison platforms, and consumer popularity lists in order to include widely accessible commercial catering services. Although meals were collected in Białystok, many of the analyzed catering companies operate in multiple regions of Poland and offer nationwide delivery. However, the providers were not selected using a formal probability-based sampling strategy; therefore, the analyzed sample should not be considered representative of the national diet catering market. The selection procedure was intended to include commonly available commercial catering services while maintaining consistent sampling conditions. From each service, three DFRs were collected on three consecutive days. Orders were placed anonymously via the official websites or mobile applications of the catering providers, in the same manner as a standard consumer order. The use of three consecutive days was intended to reduce day-to-day variability while maintaining the feasibility of laboratory analysis; however, this approach does not fully reflect weekly or seasonal variation in menu composition. Menus were selected according to the natural rotation provided by the catering services rather than being randomly assigned. Similarly, sampling days were not randomized but followed the providers’ standard menu rotation. For each provider, sampling was conducted on the same consecutive weekdays (e.g., Monday–Wednesday) to ensure consistency across providers. Additionally, only diets with a uniform energy value of 2000 kcal were analyzed, which allowed for the generation of comparable results and minimized variability resulting from differing caloric content. Standardization to 2000 kcal per day was used as a reference dietary intake level recommended for an average adult, allowing comparison with dietary guidelines for sodium intake. The selected catering services are among the most frequently chosen and widely available in Poland, as confirmed by publicly accessible rankings and diet comparison platforms. Consequently, the 40 companies included in the study represent a group of widely used providers operating in the Polish boxed diet market, making the obtained results relevant for assessing the overall sodium chloride content in such diets. The analysis covered meal sets typical of the diet for patients with Hashimoto disease *(n* = 45), the DASH diet (*n* = 45) and the low-carb diet (*n* = 30). These diet types were selected because they are commonly marketed as health-oriented or medically adapted meal plans and therefore are expected to comply with dietary recommendations, including sodium chloride restriction. The DFRs, depending on the diet type, consisted of three (*n* = 30, low-carb diet) or five (*n* = 90, diet for patients with Hashimoto disease, DASH diet) meals, with a daily calorie content of 2000 (±79.43) kcal. Differences in the number of meals per day were observed between diet types; however, the number of meals was fully confounded with diet type and therefore could not be considered as an independent variable in the analysis. In addition to the general characteristics of the sampled DFRs, the types of meals included in each set were further characterized. Depending on the diet type, DFRs typically consisted of breakfast, lunch, dinner, and two snacks (e.g., second breakfast and afternoon snack), reflecting the standard structure declared by the catering providers. For each DFR, information on the declared meal composition, including ingredients, cooking methods, and additional condiments, was collected from the manufacturers’ online menus or product labels. Portion sizes were not modified by the researchers, and all analyses were performed on meals as delivered to consumers to reflect real-life dietary exposure; however, total food mass and portion sizes were not standardized across providers, thereby reflecting real-world variability between catering services. Declared nutritional values, including energy and macronutrient composition, were recorded when available to verify compliance with the declared 2000 kcal standard. The declared menu information was not independently verified beyond comparison with delivered meals; therefore, potential discrepancies between declared and actual composition cannot be excluded.

### 2.2. Methods and Evaluation of Sodium

Each meal was separated into individual components, which were then weighed. All meals were analyzed in the form in which they were delivered to the consumer, without any modification, to preserve the original portion size and composition. From the weighed components, 20% of each was taken, and a homogenate was prepared. The use of a fixed proportion of each meal component ensured proportional representation of the entire daily food ration in the analytical sample. In the prepared samples, the sodium chloride (NaCl) content (salt equivalent), not sodium content, was determined using the Mohr method, which involves direct titration of the chloride solution with a standard 0.1 M silver nitrate solution (Sigma-Aldrich, St. Louis, MO, USA) in the presence of dipotassium chromate (VI) (Chempur, Piekary Śląskie, Poland) as an indicator in an inert environment [28]. The results were expressed as NaCl equivalent (salt content). Each sample was analyzed in triplicate, and the mean value was calculated. Analytical precision was assessed by calculating the relative standard deviation, which did not exceed 5% for replicate measurements. Homogenization was performed to ensure even NaCl distribution, and assay performance was validated against standard sodium chloride solutions (EUROCHEM BGD, Tarnów, Poland). Method precision was verified via inter-assay repeatability tests.

The NaCl content was calculated using the following equation [29]:

$$NaCl\;content\left[g/100\;g\right]=\frac{V\times N\times F\times 0.0585\times 100}{m}$$

*V*: Volume of 0.1 M AgNO_3_ used for titration;

*N*: Normality of AgNO_3_;

*F*: Factor of 0.1 N AgNO_3_;

0.0585—NaCl equivalent mass in grams per millimole;

*m*: sample weight (g).

NaCl content was converted to mass of sodium chloride in DFR.

### 2.3. Statistical Analysis

Data preprocessing and statistical analyses were performed using Python 3.12 (SciPy 1.17.1, NumPy 2.0.0, Pandas 2.2.2).

Basic statistical values such as the median (Med.), minimum (Min) and maximum (Max) values, lower (Q1) and upper (Q3) quartiles were calculated. Continuous variables are presented as individual observations and group means. Normality of distribution was assessed using the Shapiro–Wilk test, and homogeneity of variances was evaluated using Levene’s test. Differences in NaCl content between diet types (LC, DASH, H) were assessed using one-way analysis of variance (ANOVA), followed by pairwise comparisons using independent samples *t*-tests. Comparisons between two groups, including meal frequency (3 vs. 5 meals) and binary variables (soup, pickled products, one-pot meals), were performed using independent samples *t*-tests.

Effect size was estimated using Cohen’s d for all pairwise comparisons. Interpretation thresholds were as follows: 0.2 (small), 0.5 (medium), and 0.8 (large effect). Agreement between declared and measured NaCl content was evaluated using Pearson’s correlation coefficient (r). Additionally, bias was calculated as the difference between measured and declared values, and summarized as mean bias, standard deviation (SD), and the proportion of overestimated vs. underestimated values. Data visualization included violin plots combined with raw data points and mean values. Statistical significance was annotated directly on the plots using the following thresholds: ns (not significant), * *p* < 0.05, ** *p* < 0.01, and *** *p* < 0.001. Agreement between declared and measured values was additionally visualized using scatter plots with a reference line (y = x).

All tests were two-tailed, and statistical significance was set at *p* < 0.05.

## 3. Results

All NaCl values are presented as total daily intake (g/day) unless otherwise specified. The results for the NaCl content in DFRs are presented in Table 1. The amount of NaCl was calculated based on the total weight of the daily meals. The median NaCl content of all analyzed diets was 14.19 g (Q1: 10.62 g; Q3: 17.49 g). Significant differences in NaCl content in DRF were observed between diet types (ANOVA: F = 6.14, *p* = 0.0029). The highest NaCl levels were found in the Hashimoto diet, followed by the DASH diet, while the lowest values were observed in the low-carbohydrate (LC) diet.

**Figure 1:**
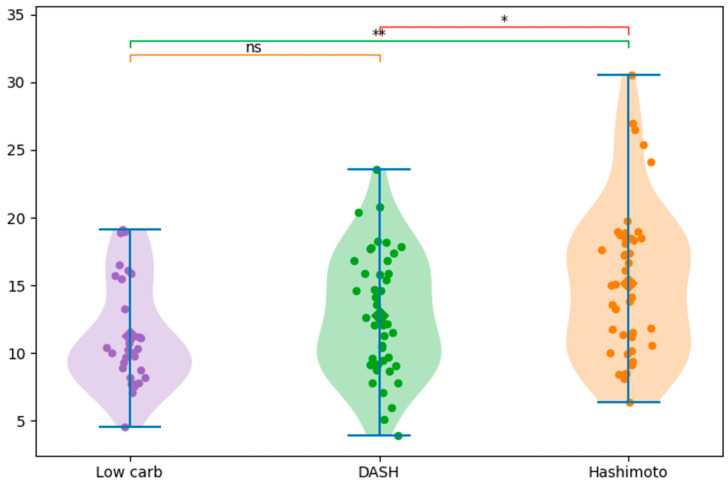
NaCl content across different diet types (Low carb, DASH, Hashimoto), ns (not significant), * *p* < 0.05, ** *p* < 0.01. Purple circles denote low-carbohydrate meals, green circles indicate DASH-compliant meals, and orange circles represent meals suitable for Hashimoto’s disease.

Pairwise comparisons showed that the Hashimoto diet contained significantly more NaCl than DASH (*p* = 0.028, d = 0.48) and LC (*p* = 0.0018, d = 0.77), indicating a moderate and large effect size, respectively. The difference between DASH and LC was not statistically significant (*p* = 0.153, d = 0.35) (Figure 1).

**Figure 2:**
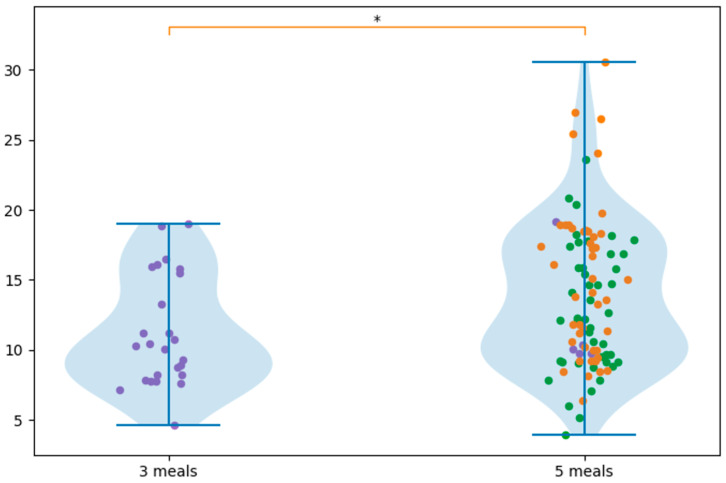
Comparison of NaCl content between meal frequency groups (3 and 5 meals). Diets consisting of 5 meals were associated with significantly higher NaCl content, * *p* < 0.05. Color coding as in Figure 1. In addition, the relationship between the presence of NaCl-rich foods in the diet: soups (*n* = 43), pickles (*n* = 8) and one-pot dishes (*n* = 23) and the overall NaCl content in DFRs was analyzed. Analysis of meal composition revealed that the presence of soup was associated with significantly higher NaCl content (*p* = 0.000073, d = 0.79), indicating a large effect size. This may be due to both excessive salting of soups, as well as the addition of bouillon cubes, concentrates or prepared seasonings, which significantly increase the total amount of NaCl. The higher NaCl content observed in certain diet types may be related to the use of processed ingredients, ready-made seasonings, and composite dishes, which are known to contribute substantially to sodium chloride intake. Furthermore, soups often contain additional ingredients such as sausage, bacon and canned vegetables, which are also important sources of NaCl in the diet. The influence of the higher NaCl content in DFRs with soups may also be due to their large volume. The presence of pickled products did not reach statistical significance (*p* = 0.0795, d = 0.65), despite a moderate effect size. Similarly, one-pot meals were not associated with a significant increase in NaCl content (*p* = 0.292, d = 0.25) (Figure 3).

Meal frequency significantly affected NaCl content (Figure 2). DFRs formulated according to the DASH diet and for patients with Hashimoto disease consisted of five meals, whereas low-carbohydrate diets consisted of three meals. Diets consisting of five meals had higher NaCl levels compared to three-meal plans (*p* = 0.0196, d = 0.68), indicating a moderate-to-large effect size. However, it should be noted that the number of meals was fully confounded with diet type in the present study design; therefore, this difference cannot be attributed to meal frequency alone and is more likely related to differences in dietary composition and total food quantity. No independent effect of meal number can be inferred. Notably, low-carbohydrate diets contained the lowest NaCl levels among all analyzed diets, despite their relatively high content of meat and fat products.

**Figure 3:**
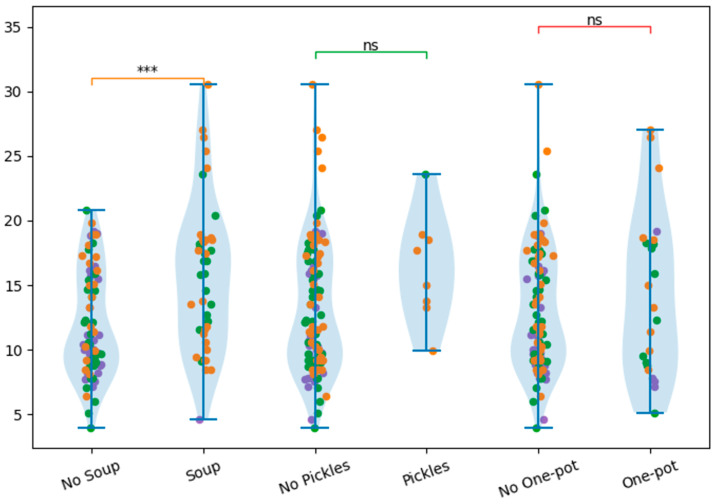
Effect of specific meal components on NaCl content: presence vs. absence of soup, pickled products, and one-pot meals. The presence of soup was associated with significantly higher NaCl levels, while pickles and one-pot meals did not show significant differences; ns (not significant), *** *p* < 0.001. Color coding as in Figure 1.

**Figure 4:**
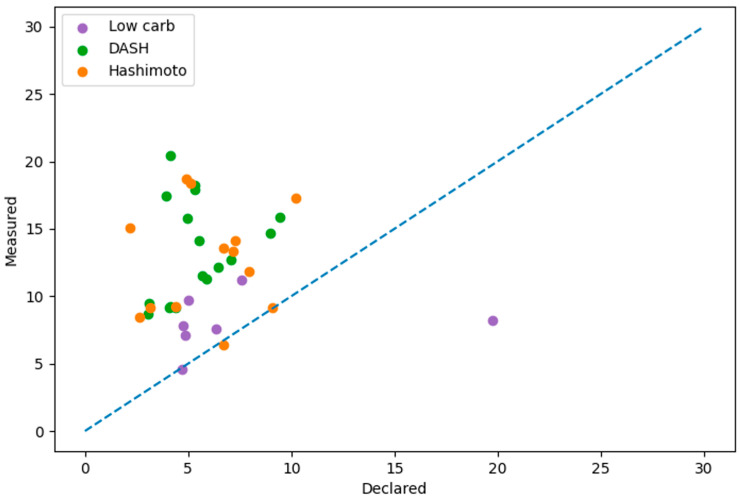
Relationship between declared and measured NaCl content. The dashed line represents perfect agreement (y = x). Deviations from the line indicate discrepancies between declared and actual NaCl values.

Only 31% of DFRs (*n* = 37) were consistent with the manufacturer’s declared NaCl content. Comparison between declared and measured NaCl content showed no significant correlation (r = −0.007, *p* = 0.966), indicating a lack of agreement between declared and actual values. Additionally, measured NaCl content was systematically higher than declared values, with overestimation occurring in 91.9% of cases (Figure 4).

## 4. Discussion

The research conducted in this study aimed to assess the NaCl content in DFRs offered by catering companies operating in Poland. The meals offered by the manufacturer as suitable for patients with Hashimoto disease and compatible with the DASH diet and the low-carb diet were analyzed. The results showed that most DFRs contained more NaCl than declared by the manufacturer (91.9%) while exceeding WHO recommended intake standards (99.2%). The median NaCl content of all diets was 14.19 g per day, which corresponds to about 5.7 g of sodium, more than twice the adequate intake (AI). According to WHO recommendations, daily salt intake should not exceed 5 g per day. Similarly, the European Food Safety Authority (EFSA) defines an AI of 2 g of sodium per day (equivalent to 5 g of salt) for adults. The findings of the present study indicate a substantial deviation from both WHO and EFSA recommendations, highlighting a lack of alignment with European public health nutrition targets. The median NaCl content exceeded these values nearly threefold, suggesting that commercially prepared daily meal plans may not align with dietary guidelines when used as the sole source of nutrition. These results may be of public health concern, particularly if such meals are consumed regularly as a primary source of nutrition. However, it should be emphasized that this study did not assess clinical outcomes or biomarkers; therefore, no causal inferences regarding hypertension or cardiovascular disease risk can be drawn. Importantly, significant differences were observed between dietary patterns, with the highest NaCl levels identified in Hashimoto diets and the lowest in low-carbohydrate diets, suggesting that sodium content may depend on the specific dietary model and menu composition rather than catering practices alone.

The discrepancy observed between analytically determined sodium content and values declared by manufacturers may further contribute to unintentional sodium overconsumption. Consumers and healthcare professionals may assume that such meals comply with dietary recommendations, while in reality sodium exposure may be substantially higher. These findings highlight the importance of improved nutritional transparency and monitoring of commercially prepared dietary plans.

From a public health perspective, the excessive sodium content observed in diet catering meals is of particular concern for individuals who rely on such services as their primary or exclusive food source over extended periods. Boxed diets are often used daily for weeks or months, which may promote sustained high sodium intake. However, the present findings should be interpreted as an assessment of nutrient exposure rather than direct evidence of health effects. Moreover, the underestimation of sodium content in catering declarations may create a false perception of dietary compliance, reducing the likelihood that consumers or healthcare professionals will identify excessive sodium intake as a modifiable risk factor. At the population level, the increasing reliance on meal delivery services may therefore contribute to maintaining or amplifying high sodium intake patterns, which may complicate public health efforts aimed at reducing population sodium intake.

Despite the growing popularity of diet catering services, research on their actual nutrient content remains limited, and most studies are based on calculated rather than analytical data. Existing literature rarely refers directly to the sodium content in such meals but confirms their high sodium levels. High sodium intake has been linked to adverse health outcomes in epidemiological studies [30,31,32]; however, the present study focused on sodium content and did not evaluate clinical effects. Increasing attention has also been given to the role of chloride ions, particularly in the context of electrolyte balance and potential effects under low potassium intake [33].

A study conducted by Gibson and Petridge in 2019 showed that all 12 meals analyzed from five Australian companies offering meal delivery kits were high in sodium (723 ± 404 to 1426 ± 688 mg per serving), exceeding the Suggested Dietary Target (SDT) by 30% [34]. Similar results were reported by McKay et al. in 2023, where all 36 analyzed meal kits exceeded recommended sodium levels [35,36]. The most comprehensive Australian study by Moores et al., analyzing 251 recipes, found that 8% exceeded daily sodium requirements, with portions providing approximately 42% of recommended intake [37].

A study conducted in Korea by Choi et al. compared sodium content in commercially prepared and homemade meals, showing no clear differences; all meals contained significant sodium levels, covering 17.1–167.3% of recommended intake [38]. In a cross-sectional study of 497 meals from United Kingdom meal kit services, NaCl content ranged from 0.2 to 6.4 g per serving (median 2.2 g), with 64.4% classified as high in NaCl and 46.5% of “health-labeled” meals exceeding recommendations [39].

In a pilot randomized controlled trial in the United States, sodium content in meal kits (433 mg breakfast; 542 mg lunch/dinner) did not exceed daily limits individually but could cumulatively approach recommended thresholds [40].

The results of the study are consistent with other studies indicating difficulty in controlling sodium content in catering meals. Unlike meal kits requiring preparation, boxed diets are fully prepared, meaning NaCl is added during production. This may explain the higher sodium levels observed compared to home-prepared meal kits. Compared to international data, Polish catering services appear to provide meals with higher sodium levels. Despite limited data, these findings indicate a need for improved meal planning and regulatory oversight, including standardized labeling and nutritional monitoring.

Excessive sodium intake is a global public health issue. In Poland, national data (2017–2020) indicate average sodium intake levels of 4887 mg/day in men and 3361 mg/day in women aged 19–64, with similarly elevated values in older adults and pregnant women [41,42]. These values significantly exceed recommendations, highlighting the need for preventive measures.

Comparison of measured and declared sodium content revealed substantial discrepancies, likely due to calculation-based labeling, lack of analytical verification, and recipe variability. As a result, consumers may unknowingly be exposed to higher sodium intake than indicated.

Given the growing popularity of catering services, further research should include a wider range of diet types and account for seasonal variability. Monitoring the nutritional quality of such meals is essential for public health.

Among the limitations of this study, several important aspects should be highlighted. Firstly, the analysis was limited to DFRs offered by selected catering companies operating in Poland, which may affect the generalizability of the findings. Secondly, the predominance of specific dietary categories, such as DASH, low-carbohydrate and Hashimoto diets, may have influenced the overall NaCl profile. Meals from each catering service were collected during three consecutive days, which may not fully capture weekly or seasonal menu variability. Furthermore, inter-provider variability was not formally analysed, as the statistical approach did not account for clustering of observations within catering companies, which may have influenced variance estimates. An additional limitation is the lack of standardization of total food mass across DFRs. Differences in portion size and total food mass across catering providers may have affected the absolute NaCl values. Another important limitation is that NaCl content was assessed at the level of total DFRs, which does not allow for precise determination of NaCl content in individual meals or specific meal categories (e.g., soups, main dishes, snacks). Although this approach reflects overall daily exposure consistent with real-life consumption patterns, it limits the ability to identify the primary dietary sources of sodium within catering diets. Future studies should therefore consider more detailed analyses at both the individual meal and meal-category levels to better characterize the main contributors to sodium intake. Variability in portion size and meal volume may have influenced the absolute NaCl values observed, particularly in diets containing high-volume items such as soups. The observed differences in NaCl content between diet types may reflect not only catering practices but also the underlying nutritional assumptions of specific dietary models, including the use of processed ingredients, composite dishes, and varying levels of dietary restriction. The study did not evaluate potential changes in sodium content during storage, transport, or reheating. Additionally, actual sodium intake may differ from the values reported, as consumers may consume additional foods or add discretionary NaCl beyond the provided meals. Finally, the comparison with manufacturers’ declarations was limited by the lack of standardized labeling regulations and inconsistent availability of nutritional data.

This study provides insight into the actual NaCl content of diets offered by catering services and highlights discrepancies between declared and measured values. The findings underscore the need for improved quality control and may support future strategies aimed at reducing dietary NaCl intake.

## 5. Conclusions

The present study demonstrated that commercially available diet catering services in Poland provide DFRs with variable sodium chloride (NaCl) content. In many cases, the measured NaCl levels exceeded recommended dietary limits, with the median NaCl content across all analyzed diets reaching 14.19 g/day, corresponding to approximately 5.7 g/day of sodium. These findings indicate that commercially prepared diet catering meals may represent a substantial source of dietary sodium chloride when used as a primary daily food source. However, due to the non-random selection of providers and the limited number of observations per service, the results should be interpreted with caution. This misalignment with established WHO and EFSA recommendations underscores the need for improved nutritional monitoring, standardized and analytically verified nutrient reporting, and enhanced quality control during meal preparation. These measures may help ensure better alignment of commercially available dietary plans with current nutritional recommendations. Future studies should include a larger and more diverse sample of catering providers and consider methodological approaches that account for variability within providers, as well as the assessment of additional nutritional parameters. It would also be valuable to include a broader range of diet types in order to help identify the most important sources potentially contributing to elevated NaCl content in meals.

## Figures and Tables

**Table 1:** NaCl content in the DFR.

NaCl Content (g)				
Type of Diets	*n*	Med.	Min–Max	Q1–Q3
Hashimoto diet	45	16.81	6.38–30.57	13.78–18.50
DASH diet	45	14.24	5.94–23.59	11.73–16.90
Low-carb diet	30	10.16	4.60–19.44	8.37–14.96
Total	120	14.19	4.60–30.57	10.62–17.49

Max—maximum, Med—median, Min—minimum, *n*—number of observations, Q1—lower quartile, Q3—upper quartile.

## Data Availability

All information will be available upon an e-mail request to the corresponding author.

## References

[1] World Health Organization Salt Reduction. https://www.who.int/news-room/fact-sheets/detail/salt-reduction.

[2] World Health Organization (2023). WHO Global Report on Sodium Intake Reduction 2023.

[3] Malczyk E., Muc-Wierzgoń M., Fatyga E., Dzięgielewska-Gęsiak S. (2024). Salt intake of children and adolescents: Influence of socio-environmental factors and school education. Nutrients.

[4] Xue X.D., Li W., Xie M.Q., Wang D.Z., Li D.D., Xin P., Zheng W.L., Jiang G.H. (2023). High sodium diet intake and cardiovascular diseases: An attributable death study in Tianjin, China. J. Clin. Hypertens..

[5] Smyth A., Yusuf S., Kerins C., Corcoran C., Dineen R., Alvarez-Iglesias A., Ferguson J., McDermott S., Hernon O., Ranjan R. (2022). Clarifying optimal sodium intake in cardiovascular and kidney (COSTICK) diseases: A study protocol for two randomised controlled trials. HRB Open Res..

[6] Chiang B.M., Ye M., Chattopadhyay A., Halezeroglu Y., Van Blarigan E.L., Abuabara K. (2024). Sodium intake and atopic dermatitis. JAMA Dermatol..

[7] Mu L., Mu L., Yu P., Xu H., Gong T., Chen D., Tang J., Zou Y., Rao H., Mei Y. (2022). Effect of sodium reduction based on the DASH diet on blood pressure in hypertensive patients with type 2 diabetes. Nutr. Hosp..

[8] Hong S., Choi J.W., Park J.S., Lee C.H. (2022). The association between dietary sodium intake and osteoporosis. Sci. Rep..

[9] Morais S., Costa A., Albuquerque G., Araújo N., Pelucchi C., Rabkin C.S., Liao L.M., Sinha R., Zhang Z.-F., Hu J. (2022). Salt intake and gastric cancer: A pooled analysis within the Stomach Cancer Pooling (StoP) Project. Cancer Causes Control.

[10] Tian Y., Lin C., Zhong H., Wu C., Wu Y., Chen B., Zhang X., Bai X., Yang Y., Wang Y. (2025). Associations and mediators of estimated sodium intake with cardiovascular mortality: Data based on a national population cohort. BMC Med..

[11] Yang Q., Vernooij R.W.M., Zhu H., Nesrallah G., Bai C., Wang Q., Li Y., Xia D., Bała M.M., Warzecha S. (2024). Impact of sodium intake on blood pressure, mortality and major cardiovascular events: An umbrella review of systematic reviews and meta-analyses. Crit. Rev. Food Sci. Nutr..

[12] World Health Organization (2016). SHAKE the Salt Habit: The SHAKE Technical Package for Salt Reduction.

[13] Martins C.A., de Sousa A.A., Veiros M.B., González-Chica D.A., Proença R.P. (2015). Sodium content and labelling of processed and ultra-processed food products marketed in Brazil. Public Health Nutr..

[14] Martini D., Strazzullo P., Serafini M., Porrini M., Pellegrini N., Angelino D. (2022). Sodium content in cereal-based products sold in Italy: How far are we from the global benchmarks?. Nutrients.

[15] Hao Z., Liang L., Pu D., Zhang Y. (2022). Analysis of sodium content in 4082 kinds of commercial foods in China. Nutrients.

[16] PMR Market Experts (2024). Dietary Catering Market in Poland 2024.

[17] Hassani Zadeh S., Salehi-Abargouei A., Mirzaei M., Nadjarzadeh A., Hosseinzadeh M. (2021). The association between dietary approaches to stop hypertension diet and Mediterranean diet with metabolic syndrome in Iranian adults. Food Sci. Nutr..

[18] Aronow W.S. (2017). Reduction in dietary sodium improves blood pressure and reduces cardiovascular events and mortality. Ann. Transl. Med..

[19] Filippou C.D., Tsioufis C.P., Thomopoulos C.G., Mihas C.C., Dimitriadis K.S., Sotiropoulou L.I., Chrysochoou C.A., Nihoyannopoulos P.I., Tousoulis D.M. (2020). DASH diet and blood pressure reduction in adults: A systematic review and meta-analysis of randomized controlled trials. Adv. Nutr..

[20] Jeong S.Y., Wee C.C., Kovell L.C., Plante T.B., Miller E.R., Appel L.J., Mukamal K.J., Juraschek S.P. (2023). Effects of diet on 10-year atherosclerotic cardiovascular disease risk (from the DASH trial). Am. J. Cardiol..

[21] Paula Bricarello L., Poltronieri F., Fernandes R., Retondario A., de Moraes Trindade E.B.S., de Vasconcelos F.A.G. (2018). Effects of the DASH diet on blood pressure, overweight and obesity in adolescents: A systematic review. Clin. Nutr. ESPEN.

[22] Chiavaroli L., Viguiliouk E., Nishi S.K., Mejia S.B., Rahelić D., Kahleova H., Salas-Salvadó J., Kendall C.W.C., Sievenpiper J.L. (2019). DASH dietary pattern and cardiometabolic outcomes: An umbrella review. Nutrients.

[23] Healthline Best Low-Sodium Meal Delivery Services. https://www.healthline.com/nutrition/low-sodium-meal-delivery#our-picks.

[24] Bankier.pl (2025). Box Diets Growing in Popularity—Clients Increasingly Include Individuals with Chronic Diseases.

[25] Chief Sanitary Inspectorate, Ministry of Health (2003). Guidelines for GHP, GMP and HACCP in Mass Catering Establishments.

[26] European Parliament and Council (2011). Regulation (EU) No 1169/2011 on the provision of food information to consumers. Off. J. Eur. Union.

[27] Orkusz A. (2022). Can you trust a box diet?. Eng. Sci. Technol..

[28] Sezey M., Adun P. (2019). Validation of Mohr’s titration method to determine salt in olive and olive brine. J. Turk. Chem. Soc. Sect. A Chem..

[29] Meija J., Michałowska-Kaczmarczyk A.M., Michałowski T. (2016). Mohr’s method challenge. Anal. Bioanal. Chem..

[30] Filippini T., Malavolti M., Whelton P.K., Vinceti M. (2022). Sodium intake and risk of hypertension: A systematic review and dose–response meta-analysis. Curr. Hypertens. Rep..

[31] Jayedi A., Ghomashi F., Zargar M.S., Shab-Bidar S. (2019). Dietary sodium, sodium-to-potassium ratio and risk of stroke. Clin. Nutr..

[32] Trakarnvanich T., Chailimpamontree W., Kantachuvesiri S., Anutrakulchai S., Manomaipiboon B., Ngamvitchukorn T., Suraamornkul S., Trakarnvanich T., Kurathong S. (2024). Effect of a low-salt diet on the progression of chronic kidney disease. J. Prim. Care Community Health.

[33] Marunaka Y. (2023). Physiological roles of chloride ions in bodily and cellular functions. J. Physiol. Sci..

[34] Gibson A.A., Partridge S.R. (2019). Nutritional qualities of commercial meal kit subscription services in Australia. Nutrients.

[35] McKay F.H. (2023). What’s in a commercial meal kit?. Public Health Nutr..

[36] McKay F.H., Zinga J., van der Pligt P. (2024). Could commercial meal kits be part of the solution to food insecurity during pregnancy?. Nutr. Diet..

[37] Moores C.J., Bell L.K., Buckingham M.J., Dickinson K.M. (2021). Are meal kits health promoting?. Health Promot. Int..

[38] Choi E., Yoon S.-W., Shin J.-A., Kim I.-H., Sung J., Ahn J.-H., Kim H.-J., Seo D.W., Lee S.-P., Lee J.-W. (2024). A comparison of the nutritional quality of ready-to-cook meals and conventional home-cooked meals in Korea. Int. J. Gastron. Food Sci..

[39] Nixon N., Ensaff H. (2024). Meal kit delivery services in the UK: An evaluation of the nutritional composition of meals. Nutrition.

[40] Hollis-Hansen K., Haskins C., Turcios J., Bowen M.E., Leonard T., Lee M., Albin J., Wadkins-Chambers B., Thompson C., Hall T. (2023). A pilot randomized controlled trial comparing nutritious meal kits and no-prep meals to improve food security and diet quality. BMC Public Health.

[41] Szostak-Węgierek D. (2020). Implementation of Comprehensive Epidemiological Studies on the Dietary Patterns and Nutritional Status of the Polish Population.

[42] Szamotulska K., Mierzejewska E., Maciejewski T., Jurczak-Czaplicka M., Fijałkowska A., Nałęcz H., Świątkowska D., Szulc J., Czach A., Honorato-Rzeszewicz T. (2020). Implementation of Comprehensive Epidemiological Studies on the Dietary Patterns and Nutritional Status of Pregnant Women.

